# Topical treatment with gallium maltolate reduces *Treponema pallidum* subsp. *pertenue* burden in primary experimental lesions in a rabbit model of yaws

**DOI:** 10.1371/journal.pntd.0007076

**Published:** 2019-01-02

**Authors:** Lorenzo Giacani, Lawrence R. Bernstein, Austin M. Haynes, B. Charmie Godornes, Giulia Ciccarese, Francesco Drago, Aurora Parodi, Sefora Valdevit, Luca Anselmi, Carlo Francesco Tomasini, Arthur M. Baca

**Affiliations:** 1 Department of Medicine, University of Washington, Seattle, WA, United States of America; 2 Department of Global Health, University of Washington, Seattle, WA, United States of America; 3 Innovation to End Neglected Diseases (IT-ENDs), East Palo Alto, CA, United States of America; 4 Gallixa LLC, Menlo Park, CA, United States of America; 5 Health Sciences Department, Section of Dermatology, San Martino University Hospital, Genoa, Italy; 6 Azienda Sanitaria 3 Genovese, Genoa, Italy; 7 Department of Medical Sciences and Infectious Diseases, Section of Dermatology, University of Pavia, Pavia, Italy; Institut Pasteur, FRANCE

## Abstract

**Background:**

Gallium is a semi-metallic element known since the 1930s to have antimicrobial activity. This activity stems primarily from gallium's ability to mimic trivalent iron and disrupt specific Fe(III)-dependent pathways, particularly DNA synthesis (due to inhibition of ribonucleotide reductase). Because of its novel mechanism of action, gallium is currently being investigated as a new antibacterial agent, particularly in light of the increasing resistance of many pathogenic bacteria to existing antibiotics. Gallium maltolate (GaM) is being developed as an orally and topically administrable form of gallium. Yaws is a neglected tropical disease affecting mainly the skin and skeletal system of children in underprivileged settings. It is currently the object of a WHO-promoted eradication campaign using mass administration of the macrolide azithromycin, an antibiotic to which the yaws agent *Treponema pallidum* subsp. *pertenue* has slowly begun to develop genetic resistance.

**Methods:**

Because yaws transmission is mainly due to direct skin contact with an infectious skin lesion, we evaluated the treponemicidal activity of GaM applied topically to skin lesions in a rabbit model of yaws. Treatment efficacy was evaluated by measuring lesion diameter, treponemal burden in lesion aspirates as determined by dark field microscopy and amplification of treponemal RNA, serology, and immunohistochemistry of biopsied tissue samples.

**Results:**

Our results show that topical GaM was effective in reducing treponemal burden in yaws experimental lesions, particularly when applied at the first sign of lesion appearance but, as expected, did not prevent pathogen dissemination.

**Conclusion:**

Early administration of GaM to yaws lesions could reduce the infectivity of the lesions and thus yaws transmission, potentially contributing to current and future yaws control campaigns.

## Author summary

Yaws is a neglected tropical disease affecting children in underprivileged countries, transmitted through direct skin contact with an active lesion. This infection, although rarely fatal, can lead to disfigurement and serious disability. The World Health Organization is currently conducting a yaws eradication effort that employs mass administration of azithromycin, an antibiotic against which the yaws pathogen has slowly begun to develop genetic resistance. Because this phenomenon has the potential to undermine the eradication effort, we investigated the antimicrobial activity of gallium maltolate, which has a novel mechanism of action, against the yaws pathogen. Our initial results show that topical application of gallium maltolate has significant treponemicidal activity, and suggest that this compound might find an application in the effort to eradicate yaws. Future studies will evaluate whether oral administration of gallium maltolate is as effective as the antibiotics currently approved for yaws treatment to clear systemic infection.

## Introduction

Yaws is a neglected tropical disease (NTD) [1,2] caused by the spirochete bacterium *Treponema pallidum* subsp. *pertenue* (*Tp pertenue*), a pathogen closely related to the syphilis agent, *Treponema pallidum* subsp. *pallidum* (*Tp pallidum*) [3]. Yaws mainly affects children less than 15 years of age, among whom the disease is spread via skin contact with an infectious early lesion [4,5], although a marginal role in transmission may be played by vector flies and non-human primates [6,7]. Similar to syphilis, untreated yaws becomes a multistage chronic disease that mainly affects the skin and skeletal system of infected individuals [2,8–11]. In contrast to syphilis, yaws is believed not to affect either the cardiovascular system or the central nervous system (CNS), and not to be vertically transmitted, even though several studies suggest that CNS, cardiovascular, and fetal involvement cannot be ruled out [12–15]. A detailed review of the early and late clinical manifestations of this disease is available elsewhere [2,8–11,16–20].

Yaws is currently reported from 14 countries in the Western Pacific, South-East Asia, and African WHO regions where, collectively, about 65,000 new cases of yaws per year have occurred since 2008 [21]. The highest disease incidence is reported in Papua New Guinea, the Solomon Islands, and Ghana [22–26]. A major anti-yaws campaign in the 1950s and 1960s by the WHO and UNICEF eradicated about 95% of the disease in 46 developing countries, causing its prevalence to drop from 50 million cases (reported in 1952) to 2.5 million cases (reported in 1964) [27]. This success induced the WHO to gradually eliminate its eradication programs, confident that the primary healthcare facilities established during the campaign would identify and eliminate the remaining cases. Lack of commitment and resources, however, led to disease resurgence in several countries [17]. In 1995, the yaws global prevalence in children was estimated to be of approximately 500,000 cases [21]. In 2013, a new campaign to achieve global eradication of yaws by 2020 was initiated by the WHO [28]. This new effort was warranted by the evidence that a single oral dose of azithromycin proved as effective as injected benzathine penicillin in curing yaws [29]. Using azithromycin could avoid the intrinsic difficulties associated with the use of penicillin, which requires an efficient cold chain and personnel able to perform injections. The use of azithromycin, however, has induced the insurgence of macrolide-resistant yaws strains whose spread could undermine the success of the ongoing campaign [30]. Additionally, even when treated with systemic antibiotics like azithromycin or penicillin, lesions might remain contagious for several hours to days post-treatment, based on studies of drug administration in the rabbit model of syphilis [31]. In this context, the application of a topical anti-treponemal agent unable to induce genetic resistance could be useful to reduce transmissibility.

Gallium (Ga) is a semi-metal that has been extensively studied as an anticancer agent, and is currently being evaluated for repurposing as a novel antimicrobial agent due to its demonstrated activity against pathogenic bacteria and its very low human toxicity [32–38]. In the early 1930s, prior to the discovery of penicillin, experiments conducted at the Pasteur Institute in Paris, France, supported the efficacy of some gallium compounds, particularly “gallium tartrate” (GaT), against *Tp pallidum* and trypanosomes [39]. These studies claimed that administration of GaT eradicated treponemes from several infected rabbits within three to four days after a single intravenous or intramuscular injection and caused the then-used Meinike reaction (a serum-induced precipitation of cholesterolized organ extracts performed to diagnose an active *Tp* infection as an alternative to the modern non-treponemal tests) to become negative [39]. The antimicrobial activity of Ga is due primarily to it acting as a non-functional mimic of Fe(III) [34,40]. Unlike Fe, which readily cycles between trivalent and divalent states, Ga is not reducible under physiologic conditions, remaining as Ga(III). By competing with Fe(III), Ga(III) can inhibit many Fe(III)-dependent biochemical activities, the most prominent being the activity of ribonucleotide reductase to synthesize DNA [34]. Recent interest in Ga compounds as antimicrobial agents [41,42] has been motivated by the need for new approaches to fight antibiotic-resistant bacteria and by the shortage of new antibiotics in the pharmaceutical pipeline.

Gallium maltolate (GaM) is currently under investigation as an orally and topically administrable form of Ga [38,40]. GaM is pH and charge neutral, and is moderately soluble in both water and lipids, making it well suited for pharmaceutical administration [40]. Locally administered GaM was effective against *Pseudomonas aeruginosa* in a mouse burn/infection model [43], it was also effective against *Staphylococcus aureus* and methicillin-resistant *S*. *aureus* (MRSA) [44] and several veterinary pathogens [45–49]. Additionally, topical GaM provided in a water/hydrophilic petrolatum emulsion was shown to have anti-inflammatory and analgesic activity in people with neuropathic pain and inflammatory conditions [38,50–53]. Here, we investigated the efficacy of topical GaM against *Tp pertenue* in a rabbit model of yaws.

## Methods

### Ethics statement

New Zealand White (NZW) rabbits were used for propagation of *Tp pertenue* and intradermal (ID) experimental infections to assess efficacy of topical GaM. Animal care was provided in accordance with the procedures described in the Guide for the Care and Use of Laboratory Animals [54] under protocols approved by the University of Washington Institutional Animal Care and Use Committee (IACUC). The protocol number assigned by the IACUC committee that approved this study is 4142–01. No investigations using human samples or humans were conducted in this study.

### Strain propagation and experimental infection

Outbred adult male NZW rabbits ranging from 3.5–4.5 Kg were purchased from Western Oregon Rabbit Co. (Philomath, OR). Rabbits were housed at 16°C to 18°C in individual cages and fed antibiotic-free food and water. Prior to entry into the study, to rule out previous infection with the rabbit syphilis agent *Treponema paraluiscuniculi*, each animal was bled and heat-inactivated sera were tested individually with both the fluorescent treponemal antibody absorption (FTA-ABS) and Venereal Disease Research Laboratory (VDRL; BD, Franklin Lakes, NJ) tests according to the manufacturer’s instructions. Only rabbits seronegative to both tests were used for either treponemal propagation or experimental ID inoculation. The *Tp pertenue* strain (Gauthier) used in these experiments was isolated in the early 1960`s in Brazzaville, Congo, from a patient’s skin lesion and provided to us by Dr. Sheila Lukehart (University of Washington), who previously received it from Dr. Peter Perine (CDC, Atlanta, GA). A 2012 frozen glycerol stock of the Gauthier strain containing 4x10^6^ *Tp* cells/ml was inoculated into the testicles of a NZW rabbit as previously described [55] and treponemes were allowed to proliferate until the animal developed an orchitis and presence of treponemal cells within testicular tissue could be assessed by dark-field microscopy (DFM) from a needle aspirate. An aliquot of the glycerol stock (100 μl) was saved for DNA extraction using the DNA Mini Kit (Qiagen, Germantown, MD) to confirm strain identity by PCR using the *tprL* gene (*tp1031*) as amplification target as previously described [56]. Briefly, the amplicon generated by *Tp pertenue* DNA (209 bp) in the *tprL* PCR assay differs in size from that originated by *Tp pallidum* and *endemicum* subspecies DNA (588 bp) due to a deletion that encompasses part of the *tprL* 5’-flanking region and ORF in the *pertenue* subspecies. Bacteria for ID inoculations of test animals were extracted from the testes of the euthanized rabbit in sterile saline supplemented with 10% normal rabbit serum (NRS). Testicular extract was collected in a sterile 15 ml tube and spun twice at 1,000 rpm (180 x g) for 10 minutes in an Eppendorf 5810R centrifuge (Eppendorf, Hauppauge, NY) to remove rabbit cellular debris. Treponemes were enumerated using DFM and percentage of motile organisms was also recorded. Extract was diluted in 10% NRS-saline to obtain approximately 7 ml of treponemal suspension at the desired concentration (10^7^ cells/ml). Test (n = 4) and control rabbits (n = 2) were injected ID with 100 μl of treponemal suspension (containing 10^6^ treponemes) in 10 sites on their clipped backs. The skin was marked with permanent ink one inch below each injection site to facilitate location of the lesions. Following ID injection, treponemal motility was assessed again using DFM to ensure that the time elapsed between harvest and ID inoculation did not affect pathogen viability. After ID inoculation, rabbit backs were clipped daily to allow monitoring of lesion development and surgical procedures to collect lesion biopsies and aspirates. For the purpose of antimicrobial testing, ID infection is preferable to IT infection because skin lesions can be readily aspirated for DFM examination to assess the presence of *Tp* cells and evaluate treatment efficacy.

### Gallium maltolate application

A 0.5% w/v GaM cream was provided by Gallixa (Menlo Park, CA) along with the carrier alone (an emulsion of water and hydrophilic petrolatum). Test animals were divided into three groups (Groups 1–3), each group containing two rabbits. Groups 1 and 2 received GaM twice a day every 12 hours. Group 1 rabbits began treatment at the first clinical evidence of infection by DFM analysis of lesion aspirates (day 4 post-inoculation), while Group 2 rabbits received GaM when lesions had become clearly indurated (day 14 post-inoculation). Administration at day 4 post-inoculation, was performed to evaluate GaM ability to prevent lesion progression, while application at day 14 post-infection aimed at evaluating GaM ability to reduce treponemal burden faster than in control animals and accelerate lesion healing. Group 3 rabbits received an equal amount of carrier only from day 4 post-inoculation. Treatment consisted in applying 250 μL of either GaM or carrier on top of each lesion, followed by gentle manual spreading to ensure uniform coverage of the whole lesion. Following application, animals were monitored for a few minutes to ensure that they would not remove the ointment, and were then taken back to their cages.

### Monitoring of lesion development and evaluation of treponemal burden by microscopy and RT-qPCR

**Fig 1:**
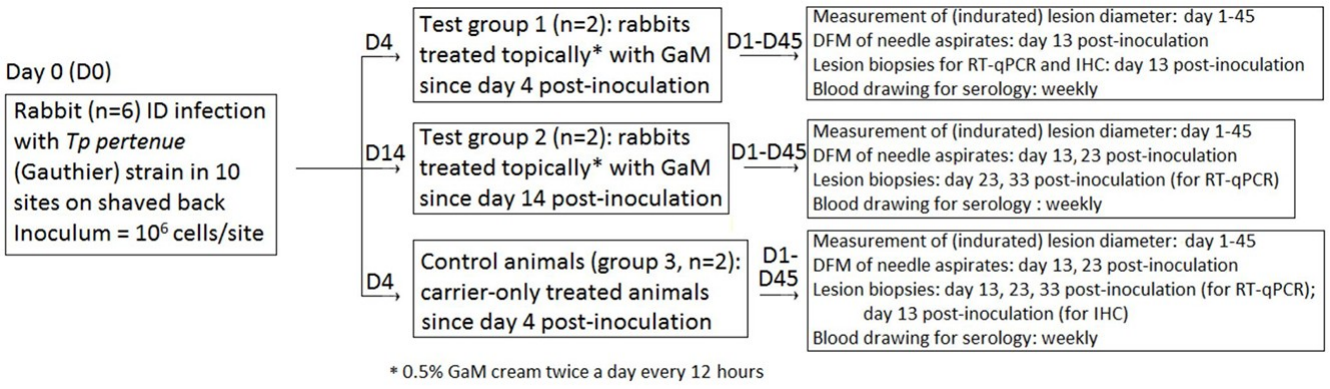
Flowchart of the experimental design and procedures.

Development of skin lesions at injection sites was monitored in all experimental subjects by measuring diameter of indurated lesions each day, after shaving the animals and prior to the first application of GaM or carrier. Appearance of lesions at distant sites, due to pathogen hematogenous dissemination from the primary injections sites, was also monitored. Treponemal burden within primary lesions was first assessed by performing DFM analysis of lesion needle aspirates of all lesions at day 13 post-inoculation. At day 23 post-inoculation, a second set of aspirates was obtained from Group 2 (GaM-treated since day 14 post-inoculation) and Group 3 (carrier-treated control) rabbits from all lesions (except those that were previously biopsied). Approximately 100 fields per slide were examined and the treponeme number was recorded. Two lesion biopsies were obtained at day 13 post-inoculation using a 4 mm biopsy punch from each of Group 1 (GaM-treated since day 4 post-inoculation) and Group 3 (control) animals for evaluation of treponemal burden by real-time amplification of *Tp* mRNA (see below). Two additional biopsies were obtained at day 25 and day 33 post-inoculation from each animal in Group 2 (treated from day 14) and Group 3 (control). Inoculation sites to be biopsied were selected randomly. A flowchart describing the experimental design is provided in Fig 1. In all cases, biopsy samples were minced with a sterile scalpel immediately after collection and further homogenized in 400 μl of phenol-based TRIzol buffer (Life Technologies, Santa Clara, CA) using a disposable plastic pestle. Samples were stored at -80°C until use. Total RNA from biopsies was obtained according to the TRIzol extraction protocol. Extracted RNA was treated with DNaseI to obtain DNA-free RNA as previously described [57]. Reverse transcription into cDNA was performed with the Superscript III First-Strand Synthesis System (Life Technologies) using random primers according to the manufacturer's instructions. Message quantification was performed using an established relative quantification method that targets the mRNA for the treponemal 47 kDa lipoprotein (encoded by the *tp0574* gene) and that normalizes the *tp0574* signal to the message for the rabbit housekeeping gene Hypoxanthine-Guanine Phosphoribosyl Transferase (HPRT) [57]. Primer sequences and real-time amplification conditions for both targets, as well as details on plasmid standard preparation, were previously published [57,58]. Data from message quantification and treponemal counts from aspirates were analyzed with Student’s unpaired two-tailed t-test and significance set at *p*≤0.05. All animals were bled weekly for serology (FTA-ABS and VDRL), to assess seroconversion and confirm establishment of infection. Animals were euthanized 45 days post-inoculation after serological evidence demonstrated that all had become infected. Serological assays were performed on the same day for all sera collected at a given time point, to minimize test-to-test variability. The technologist performing the assays was blinded to the treatment status of the animals from which the samples were collected.

**Immunohistochemistry (IHC).** At day 13 post-inoculation a 4-mm lesion biopsy was taken randomly from each animal in Group 1 and Group 3. Two additional biopsies from disseminated skin lesions that appeared in one of the Group 1 rabbits were also taken. All biopsies were fixed in 10 ml of 10% neutral buffered formalin (NBF) at room temperature for approximately 72 hours and then transferred to 70% ethanol and stored at 4°C until paraffin embedding and sectioning. For embedding, biopsies were transferred back into 4% NBF (PanReac Applichem, Barcelona, Spain) for 3 hrs (2 x 1.5 hr passages). Subsequent sample processing was performed in a Leica ASP300 instrument (Leica Biosystems, Wetzlar, Germany). Samples were incubated in water for 10 min, and then transferred in 80% ethanol for 1 hr, in 96% ethanol for a total of 2 hrs (2 x 1 hr passages), and in absolute ethanol for 2 hrs (2 x 1 hr passages). Following dehydration, samples were transferred into paraffin solvent (Histo-Clear, National Diagnostics, Atlanta, GA) for 2 hrs (2 x 1 hr passages), followed by three passages of 1 hr each in liquid paraffin at 58°C (Paraplast X-tra, Millipore-Sigma, St. Louis, MO). From these samples, 3-μm sections were cut, placed on a heath block at 65°C for a total of 20 min to allow tissue adherence to the slide, and then stored at room temperature. For IHC procedures, silane-treated slides were used to further improve tissue adherence, and tissue sections were stored at 37°C. For hematoxylin and eosin (HE) staining, deparaffinized and rehydrated sections were placed in hematoxylin solution for 8 min and then rinsed for 3 min with tap water. Eosin staining was carried on for 1 min prior to washing under tap water for 5 minutes. Dehydration was obtained by passage in 96% ethanol for 4 min, followed by 2 passages in 100% ethanol for 2 and 3 min, respectively, and 2 passages in Histo-Clear for a total of 3 min. Sections were mounted using an acrylic resin (Eukitt, Orsatech, Gmbh), taking care to leave no bubbles during the process. Slides were left to air-dry overnight before being analyzed. For specific immunostaining, slides were first heated at 65°C for 1 hr, and then deparaffinized in EX-Prep solution (Roche Diagnostics, Indianapolis, IN) at 72°C. Cell conditioning was performed by applying ULTRA CC1 solution (Roche diagnostics) for a variable time (20–36 min, depending on the primary antibody) to correct epitope alteration due to fixation of the tissue sections. Polyclonal anti-CD4, -CD8, and -CD20 primary antibodies (Roche Diagnostics) were used at 1:100 dilution, and slides were incubated for 16 min (anti-CD4 and -CD8) or for 12 min (anti-CD20 antibodies). To avoid evaporation, tissue sections were covered with ULTRACS liquid coverslip (Roche Diagnostics) following application of the primary antibody. Primary antibody was removed by washes with a Tris-based buffer solution (Reaction buffer, pH 7.6; Roche Diagnostics). Reagents provided in the Ultra View Universal DAB (3,3’ diamino-benzidine) Detection kit (Roche Diagnostics) were used according to the manufacturer`s instruction for detection of primary antibody binding. Slides were then rinsed with water and counterstained with hematoxylin for 12 minutes. Tissue sections were dehydrated using two 5-min rinses with 96% ethanol followed by two 5-min rinses in absolute ethanol, and two 10-min washes with Histo-Clean. Prior to reading, coverslips were mounted on slides using the Eukitt acrylic resin and air-dried prior to being read.

## Results

### Lesion development and treponemal burden

**Fig 2:**
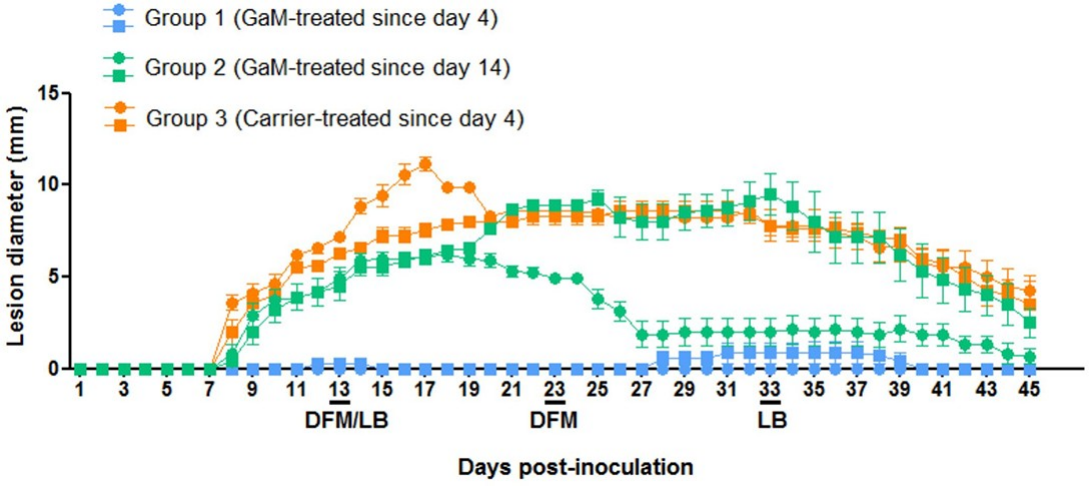
Measurement of lesion diameter in treated and control rabbits. Lesion diameter (mm) was measured starting 24 hours post-inoculation (day 1) up to day 45. Group 1 rabbits (n = 2, blue) were treated since day 4 post-inoculation, while Group 2 animals (n = 2, green) were treated since day 14. Control animals (orange) received only carrier (an emulsion of water and hydrophilic petrolatum) since day 4 post-inoculation. Days 13 and 23 are underlined to indicate the days on which DFM of lesion aspirates was performed and/or lesion biopsies (LB) were obtained. Within in each group, the lesion diameter from each individual rabbit is designated with a dot or square.

**Fig 3:**
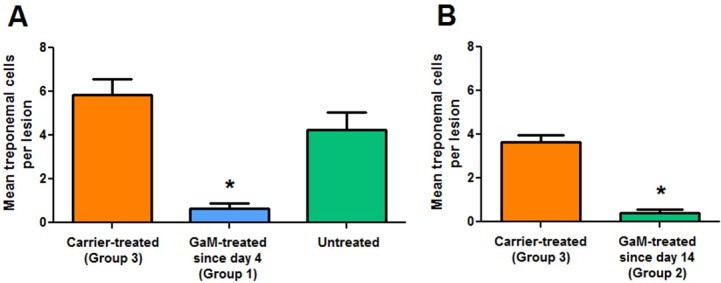
DFM analysis of lesion aspirates. Panel A shows pathogen burden at day 13 post-infection in Group 1 animals (treated with GaM since day 4 post-infection) compared to carrier-treated animals (Group 3) and untreated animals (which started treatment the following day and were then identified as Group 2). The untreated animals started GaM treatment on day 14 post-infection. Panel B shows pathogen burden at day 23 post-infection in Group 2 animals (treated with GaM since day 14 post-infection) compared to carrier-treated controls (Group 3). Asterisks indicate statistical significance (p<0.05).

Measurements of lesion diameter as a function of time (Fig 2) showed that in animals treated with GaM since day 4 post-inoculation, lesion development was significantly attenuated compared to controls (Group 3) or to animals treated since day 14 post-inoculation. Most lesions form Group 1 animals failed to develop into indurated papules and enlarge, but rather remained flat, although generally erythematous. Compared to controls, only one of the Group 2 rabbits that initiated treatment at day 14 post-inoculation showed a significant decrease in lesion diameter. Assessment of treponemal burden by DFM on lesion aspirates obtained at day 13 (Fig 3A) and day 23 (Fig 3B) post-inoculation showed that compared to controls and untreated animals, treponemal burden in lesions from GaM-treated animals was significantly reduced (p<0.05), while carrier-treated animals and untreated did not show significant differences in number of treponemes counted. Analysis of treponemal burden performed at day 23 post-inoculation showed that overall significantly fewer treponemes could be found in lesions from rabbits that started treatment at day 14 post-infection (Group 2) compared to controls. Table 1 summarizes the DFM results for each animal in each group, together with the results of FTA-ABS and VDRL tests following experimental infection. Notably, treated animals seroconverted approximately a week later than control animals, which is consistent with the reduced treponemal burden due to GaM.

**Table 1:** DFM, FTA-ABS, and VDRL results.

Rabbit # (Group #)	DFM results Number of positive lesions/total (Mean number of treponemes/lesion)	FTA-ABS^1^ results (reactivity)	VDRL^1^ results			Disseminated lesions by day 45 post-inoculation
Day 13 post-inoculation						
#125 (Group 1) #126 (Group 1)	4/10 (0.7) 3/10 (0.6)	Day 41 (+3) Day 41 (+1), day 45 (+3)	Day 45 Day 45			Yes Yes
#127 (Group 2) #129 (Group 2)	7/10 (3.8) 8/10 (4.7)	Day 41 (+2) Day 41 (+3)	Day 45 Day 41			Yes Yes
#122 (Group 3) #128 (Group 3)	10/10 (7.1) 8/10 (4.6)	Day 36 (+1), day 41 (+2) Day 36 (+1), day 41 (+4)	Day 36 Day 36			Yes Yes
Day 23 post-inoculation						
#127 (Group 2) #129 (Group 2)	2/10 (0.2) 3/10 (0.6)					
#122 (Group 3) #128 (Group 3)	7/7 (2.3) 7/7 (2.8)					

^1^Day post-infection at which seroconversion was first observed.

**Fig 4:**
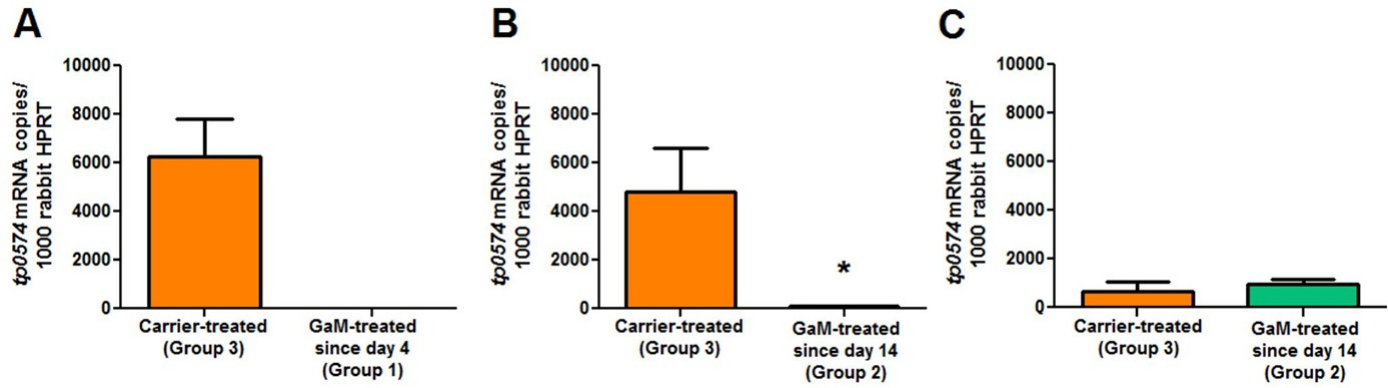
Quantification of treponemal *tp0574* mRNA in lesion biopsies. Treponemal burden in lesion biopsies harvested at day 13 post-inoculation from Group 1 and Group 3 animals (A), day 23 post-inoculation in Groups 2 and 3 rabbits (B), and at day 33 post-inoculation in Groups 2 and 3 rabbits (C). *tp0574* copy number is normalized to 100 copies of rabbit HPRT message. Asterisk indicates statistical significance (p<0.05).

Treponemal burden in primary lesions was further assessed using *Tp* mRNA quantification normalized to the rabbit housekeeping gene HPRT. Message quantification at day 13 post-inoculation showed that no treponemal mRNA was detected from Group 1 rabbit lesions compared to controls (Fig 4A). At day 23 post-infection, significantly less (p<0.05) *Tp* mRNA was detected in lesions from Group 2 rabbits compared to controls, while no difference was seen between these rabbits and the control ones at day 33 post-inoculation. By the end of the experiment (day 45 post-inoculation) all animals had developed erythematous disseminated skin lesions. Analysis of needle aspirates from a small subset of disseminated lesions revealed the presence of treponemes by DFM (not shown).

### Histological analysis of lesion biopsies

**Fig 5:**
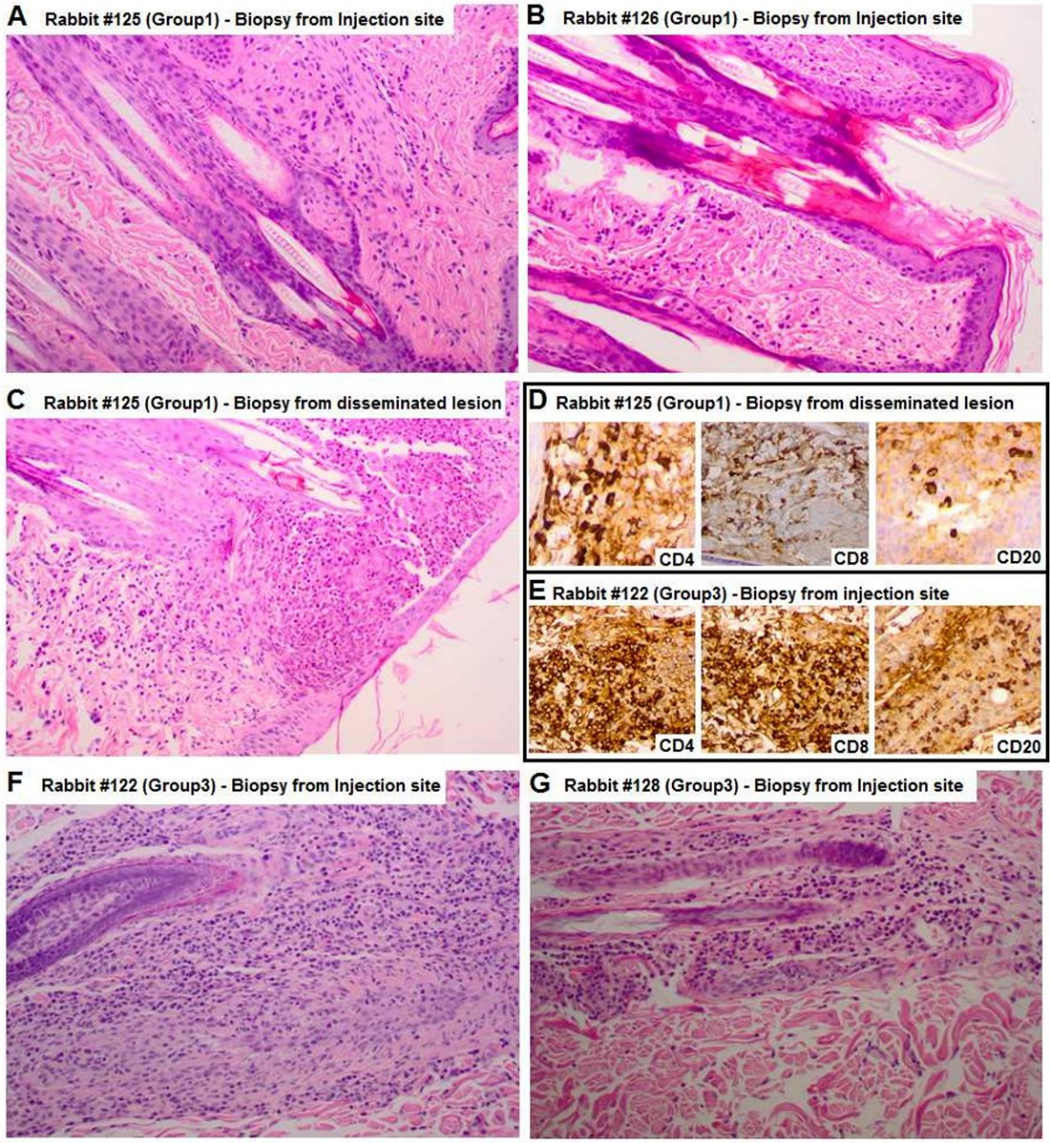
Histological analysis of lesion biopsies. **Hematoxylin-Eosin.** (A, B) Hematoxylin/eosin (HE) straining of biopsies obtained from Group 1 rabbits (#125 and #126, treated since day 4 post-inoculation). (C) HE staining of biopsy from a disseminated lesion of one of the Group 1 rabbits. (D, E) CD4/CD8/CD20 specific staining for the disseminated lesion biopsy of rabbit #125 (Group 1) and the injection site biopsy from control rabbit #122 (Group 3, treated since day 4 with carrier only). (F, G) HE staining of biopsies from lesion developed at the injection sites of Group 3 rabbits (#122, and #128), treated with carrier only.

Both biopsies obtained from Group 1 rabbits showed the presence of very modest inflammatory infiltrates and absence of damage to follicles (Fig 4A and 4B), nearly like normal skin. Histological analysis of a disseminated lesion biopsy from one of the Group 1 rabbits (Fig 5C and 5D) showed a rich infiltrate of inflammatory cells, particularly eosinophils, and comparable amounts of CD4 and CD8 T-lymphocytes, plus B-lymphocytes (CD20 cells) and plasma cells, as well as follicular inflammation and intra-follicular abscesses (Fig 5C and 5D). Analysis of a second disseminated lesion from the same animal also showed extensive follicular inflammation and an elevated number of eosinophils, although the lymphocyte component could not be evaluated due to a scarcity of cells (not shown). Also, biopsies from carrier-treated animals showed a significant inflammatory infiltrate composed of eosinophils, lymphocytes (CD4, CD8, and CD20), and plasma cells (CD138+ cells) (Fig 5E–5G). Biopsies from control animals also showed elevated numbers of histiocytic cells and blood vessels with a thickened endothelium (Fig 4F and 4G).

## Discussion

In a rabbit model of yaws, GaM demonstrated treponemicidal activity against *Tp pertenue* is consistent with the previously claimed activity of gallium against *T*. *pallidum* [39]. When applied 4 days after inoculation, GaM significantly attenuated lesion development and decreased treponemal burden within lesions developed at the inoculation sites (assessed by both DFM and quantification of *Tp*-specific mRNA), indicating that GaM is bactericidal when applied locally. Secondary lesions distal to the treated inoculation site still appeared, with detectable treponemes by DFM and a histopathological picture consistent with inflammation, indicating that the predominant effect of topical GaM is localized to the area where applied. Treponemal systemic dissemination from the site of exposure is known to be rapid [59], and topical antimicrobial application was not expected to lead to disease eradication. When applied 14 days after inoculation, GaM promoted lesion healing in one rabbit but not the other. Both of those rabbits, however, showed a significant decrease in treponemal burden by DFM and qPCR. These limited data suggest that yaws lesions that have progressed from the initial erythematous stage may still benefit from GaM treatment. This small pilot study provides justification for conducting larger studies to further investigate GaM as a possible therapeutic agent for yaws. As part of its 2012 plan to overcome the global impact of neglected tropical diseases, the WHO set 2020 as the target year for yaws eradication [60]. The success of the previous anti-yaws campaigns conducted in the 1950`s [27] was due in large part to the efficacy of penicillin against the yaws pathogen, and the apparent inability of this pathogen to develop penicillin resistance. The reasons behind this inability to develop resistance are being elucidated only now [61]. Although penicillin is still the treatment of choice for yaws, in 2012 the yaws eradication strategy was revised to make single-dose oral azithromycin the treatment of choice for mass drug administration. This decision was driven by the difficulties associated with penicillin delivery, which requires a cold chain and trained personnel *in loco*. Similar to what has happened with syphilis treatment, the introduction of azithromycin has induced the emergence of macrolide-resistant yaws strains, with five epidemiologically related cases in Lihir island, Papua New Guinea [30]. The spread of these azithromycin-resistant strains could therefore undermine the current eradication goal, and make macrolides a decreasingly effective treatment option for yaws, similar to the situation with syphilis. GaM appears to be an attractive alternative treatment for yaws due to its novel mechanism of action (acting as a non-functional mimic of Fe(III), inhibiting bacterial DNA synthesis, for which the development of resistance appears unlikely), and its ability to be administered both locally (topically) and systemically (orally). Because azithromycin clears treponemes from dermal lesions slower than does penicillin [31], we hypothesize that in azithromycin-treated patients, concurrent topical GaM treatment of the yaws lesion during dressing changes may accelerate pathogen clearance from the lesion and consequently accelerate wound healing. This would allow a yaws patient to return to a normal life more quickly as well as decrease the infectivity of the lesion. If shown effective, co-administration of GaM with azithromycin as a “protective drug” could help delay appearance and spreading of genetic resistance to macrolides, greatly extending the time window in which azithromycin could be effectively used for yaws eradication. Systemically administered gallium was claimed in the past to have anti-treponemal activity in a rabbit model of syphilis [39]. We are planning to investigate the efficacy of orally administered GaM in our rabbit model of yaws. GaM has already completed several Phase 1 clinical trials in humans, where high safety and tolerability was demonstrated for this compound [38,62]. In those trials, GaM was administered orally to cancer patients at doses of as much as 3500 mg/day for repeated 28-day cycles; no dose-limiting or other significant toxicity was reported [63]. The topical GaM dose that would be applied to yaws lesions would be about a thousandth of the highest apparently safe oral dose, so the safety is expected to be high.

### Limitations

The major limitation of this study was the small number of laboratory animals used, which did not allow us to make clear conclusions on the efficacy of GaM in accelerating lesion healing when administered to indurated lesions (Group 2 animals). The promising results of this pilot study, however, suggest that the experiments described here should be repeated with groups of at least 8 rabbits each, according to power/sample size calculations. Furthermore, although topical GaM application was shown to have treponemicidal activity, as expected it did not prevent pathogen dissemination and establishment of the infection, even in animals that started treatment as soon as day 4 post-challenge. Our study did not address the efficacy of systemic GaM in eradicating experimental yaws. Additional studies on oral administration of GaM alone or in combination with conventional antimicrobials will need to be performed to fill this knowledge gap. Lastly, a large inoculum (10^6^ cells/injection site) was used to induce lesion development within an acceptable experimental time-frame and to obtain samples in which the treponemal burden could be quantified. Very likely, during natural human infection, significantly fewer treponemes pass the epithelial barrier to cause disease. The use of large inocula has previously been used to evaluate the effectiveness of azithromycin against *Tp pallidum*, and was not shown to be a confounding factor, and we have no reason to believe it could be in our studies either. Gallium has previously been shown to be effective against many microorganisms *in vitro* and in animal models [41,42]. This study is the first to extend these observations to *Tp pertenue* and to report the use of GaM as a topical treatment for yaws. Our results suggest that this compound could be useful as a topical anti-treponemal agent, and justifies further research into the use of GaM as both a topical and an oral agent, alone and/or in combination with other antimicrobials to assess its full potential as a novel anti-yaws compound.
